# The Prevalence, Risk Factors, and Short-Term Health Outcomes of Delirium in Patients Admitted to a Nephrology Ward in Eastern Europe: An Observational Prospective Cohort Study

**DOI:** 10.3390/jcm14207303

**Published:** 2025-10-16

**Authors:** Florica Gadalean, Ligia Petrica, Oana Milas, Flaviu Bob, Florina Parv, Cristina Gluhovschi, Anca Suteanu-Simulescu, Lavinia Marcu, Mihaela Glavan, Silvia Ienciu, Raluca Kigyosi, Alina Stanigut

**Affiliations:** 1 Department of Internal Medicine II, Division of Nephrology, “Victor Babes” University of Medicine and Pharmacy Timisoara, Romania, Eftimie Murgu Sq. No. 2, 300041 Timisoara, Romania; gadalean.florica@umft.ro (F.G.); petrica.ligia@umft.ro (L.P.); bob.flaviu@umft.ro (F.B.); gluh@umft.ro (C.G.); anca.simulescu@umft.ro (A.S.-S.); lavinia.balint@umft.ro (L.M.); patruica.mihaela@umft.ro (M.G.); ioana.ienciu@umft.ro (S.I.); 2 County Emergency Hospital Timisoara, 300723 Timisoara, Romania; parv.florina@umft.ro; 3 Center for Molecular Research in Nephrology and Vascular Disease, Faculty of Medicine, “Victor Babes” University of Medicine and Pharmacy Timisoara, Romania, Eftimie Murgu Sq. No. 2, 300041 Timisoara, Romania; 4 Center for Translational Research and Systems Medicine, Faculty of Medicine, “Victor Babes” University of Medicine and Pharmacy Timisoara, Romania, Eftimie Murgu Sq. No. 2, 300041 Timisoara, Romania; 5 Centre for Cognitive Research in Neuropsychiatric Pathology (Neuropsy-Cog), Faculty of Medicine, “Victor Babes” University of Medicine and Pharmacy Timisoara, Romania, Eftimie Murgu Sq. No. 2, 300041 Timisoara, Romania; 6 Department of Internal Medicine II, Division of Cardiology, “Victor Babes” University of Medicine and Pharmacy Timisoara, Romania, Eftimie Murgu Sq. No. 2, 300041 Timisoara, Romania; 7 Department of Geriatrics and Gerontology, “St. Luca” Hospital Bucharest, Street Șoseaua Berceni, No.12, 041915 Bucharest, Romania; raluca-cristiana.kigyosi0925@rez.umfcd.ro; 8 Clinical Medical Disciplines Department, Medicine Faculty, “Ovidius” University of Constanta, 1 Universitatii Street, 900470 Constanta, Romania; alina.stanigut@365.univ-ovidius.ro; 9 Nephrology Department, “Sf. Apostol Andrei” Emergency County Hospital, 145 Tomis Blvd., 900591 Constanta, Romania

**Keywords:** delirium, in-hospital mortality, nephrology, prevalence, risk factors

## Abstract

**Background/Objectives:** To date, delirium is considered one of the most frequent acute neuropsychiatric syndromes among hospitalized populations, although there is a lack of data regarding its frequency and predictors in nephrological patients. The aims of this study were to investigate the prevalence of and the risk factors for delirium and to evaluate the association between delirium and short-term clinical outcomes, including the length of stay (LOS) and in-hospital mortality rate among patients from the nephrology unit of a tertiary university hospital in Eastern Europe. **Method:** A cohort of 942 patients admitted between January 2023 and December 2023 were enrolled in a prospective observational study. Delirium was diagnosed by a psychiatrist during hospitalization. The endpoint was defined as hospital death or hospital discharge. **Results:** In the studied group, the median age was 65 years, and 519 (55.09%) patients were males. The prevalence of delirium was 5.41% (51/942 patients). The patients with delirium had a significantly longer LOS (11.96 days vs. 8.86 days, *p* = 0.007) and a significantly higher in-hospital mortality rate (47.05% vs. 14.36%, *p* < 0.001) compared to the patients without delirium. The independent predictors of delirium were as follows: age (OR = 1.029; 95%CI: 1.002–1.056; *p* = 0.034), history of stroke (OR = 3.493; 95%CI: 1.849–6.598; *p* < 0.001), alcohol abuse (OR = 4.728; 95%CI: 1.968–11.359; *p* = 0.001), and AKI stages 2 and 3 (OR = 2.175; 95%CI: 1.152–4.105; *p* = 0.017). From a time-to-event analysis, delirium was associated with increased mortality (HR = 2.77; 95%CI: [1.79 to 4.29]; *p* < 0.001). Moreover, delirium was independently associated with increased in-hospital mortality (OR = 1.666; 95%CI: 1.069–2.597; *p* =0.024). **Conclusions:** Among nephrological patients, age, alcohol abuse, history of stroke, and AKI stages 2 and 3 were independent risk factors for delirium. Delirium significantly increased the LOS and in-hospital mortality.

## 1. Introduction

Delirium is a neurocognitive syndrome characterized by an acute alteration in attention and consciousness, and is associated with cognitive deficits. This alteration develops over a short period and fluctuates in its presence and severity over the course of 24 h [1]. Delirium can be triggered by numerous factors, such as an acute medical illness (like liver failure, sepsis, hypoglycemia, and stroke), medication use or withdrawal, surgery, or trauma. Most causes begin outside the brain, though delirium with primary nervous system causes, such as stroke, has also been described [1,2,3].

Delirium is a common disorder among hospitalized patients [3]. Its prevalence ranges widely, from 5% to 54%, depending on the patient’s characteristics, such as age, previous neurodegenerative disorders, and frailty [4,5]. Moreover, the healthcare setting and kind of screening instruments affect the estimations [3].

Individuals with delirium suffer poor short- and long-term outcomes, which include prolonged hospital stays [6,7,8], increased mortality [8,9,10,11,12], and increased rates of institutionalization [13]. Moreover, delirium generates functional and cognitive decline with an almost 9-fold increase in the risk of onset of dementia in the first year after a delirium episode [14].

In addition, the financial burden of delirium on patients exists worldwide and can be substantial. Annually, in the United States, the medical costs for delirium vary from USD 143 billion to USD 152 billion [15]. In Australia, the healthcare expenses associated with delirium amount to approximately AUD 8.8 billion annually [16], while in 18 European countries combined, they surpass USD 182 billion per year [4].

Although delirium is associated with significant negative clinical and economic consequences, it often remains unrecognized and easily neglected [3,4,17]. The data from the literature shows that the rate of delirium diagnoses by physicians is estimated to be 7–57% [18], and by nurses to be 26–83% [4,5]. As delirium is frequently underdiagnosed, the current delirium guidelines routinely use highly sensitive and highly specific delirium screening tools [19]. Hence, delirium may be preventable in 30% to 40% of patients; its early detection has a very important role in improving a patient’s outcome, reducing the length of hospital stay (LOS), and reducing healthcare costs [4].

Most delirium research focuses on specific patient groups, such as those who are mechanically ventilated, elderly, have psychiatric conditions, or undergo surgery [6,8,20,21,22,23]. To date, only a few studies have investigated the occurrence, risk factors, and consequences of delirium in patients with kidney disorders. These studies have mainly included patients with critical illness and acute kidney injury (AKI) admitted to Intensive Care Units (ICUs) [7,24], and patients with end-stage chronic kidney disease (ESKD) undergoing hemodialysis (HD), respectively [25,26].

As far as we know, there is no study that has evaluated the development of delirium and its consequences among patients hospitalized in a nephrology ward. We therefore conducted a prospective observational study evaluating delirium in a nephrology unit of a tertiary university hospital in Eastern Europe.

The aims of this study were to investigate the prevalence of and the risk factors for delirium and to evaluate the association between delirium and the short-term clinical outcomes, including the LOS and in-hospital mortality rate.

## 2. Materials and Methods

This prospective observational study included patients aged 18 years or above who were admitted to the Department of Nephrology of the County Hospital Timisoara, Romania, between January 2023 and December 2023. The County Emergency Hospital of Timisoara is a tertiary teaching hospital which provides health care to the western region of Romania, which has a population of over 2,000,000 residents.

Patients who were unconscious or who suffered from prior severe cognitive impairment that made verbal communication impossible were excluded. Also, we excluded subjects who had fewer than two serum creatinine (SCr) measurements during hospitalization, and/or were hospitalized for less than 48 h, and patients younger than 18 years old. The patients were followed up with until discharge, transfer to other departments, or death.

### 2.1. Ethics Statement

The County Emergency Hospital of Timisoara Ethical Committee (Board of Human Studies) approved the protocol (approval number 1/3 January 2023), and all patients gave informed consent. The study was conducted in conformity with Strengthening the Reporting of Observational Studies in Epidemiology (STROBE) recommendations [27].

### 2.2. Variables

The data regarding socio-demographics and comorbidities were collected from the GPs’ files. The following data were recorded: age; gender; smoking status; alcohol abuse; and comorbidities, including diabetes mellitus (DM), arterial hypertension (AH), coronary artery disease (CAD), congestive heart failure (CHF), previous stroke, vascular dementia, peripheral vascular disease (PVD), pre-existing chronic kidney disease (CKD), chronic liver disease, chronic pulmonary disease, and depression. Acute kidney injury (AKI) was defined and staged according to the serum creatinine criteria of the Kidney Disease Improving Global Outcomes (KDIGO) Guidelines [28]. Information on pre-admission medication was not collected due to its identification in only a subset of participants.

The baseline kidney function was evaluated using the CKD-EPI creatinine equation [29]. The baseline SCr was estimated as the mean outpatient SCr 7–365 days before hospitalization [30], or if not available, we utilized the lowest SCr value during hospitalization as the baseline SCr [31].

All patients were evaluated for delirium if their clinical presentations indicated possible signs of delirium (e.g., restlessness, disorientation, confusion, agitation, sleep disturbances, etc.) and a psychiatric consultation was requested to confirm the diagnosis of delirium according to the *Diagnostic and Statistical Manual of Mental Disorders, 5th edition* [1].

## 3. Outcomes

The following two clinical outcomes were assessed: (1) the prevalence of and risk factors for delirium development and (2) the association between delirium and the following outcomes—LOS and death during hospitalization.

### Statistical Analysis

The continuous variables were reported as the means ± standard deviations for the normally distributed data and as medians (interquartile ranges) for the continuous variables without a Gaussian distribution, while the categorical variables were presented as numbers and percentages. The differences between the studied groups were analyzed with Student’s *t*-test (means, Gaussian variables) and with the Mann–Whitney U test (medians, non-Gaussian variables), respectively, with a chi-square test or Fisher’s exact test (proportions). The analysis of survival was performed using the Hazard Ratio (HR) method and was represented using Kaplan–Meier diagrams. The log-rank (Mantel–Cox) test was used to evaluate the differences between the survival curves.

A univariable logistic regression analysis was performed to assess the association of each risk factor with incident delirium. Then, the parameters that were statistically significant (*p* < 0.05) in the univariable regression analysis were included in a multivariable model using the stepwise backward method. At each step, the parameter with a *p* value > 0.05 was removed from the model, and we obtained a final model composed only of statistically significant variables (*p* < 0.05). In the multivariable regression analysis, the association between every one risk factor and incident delirium was evaluated by the Wald test.

To assess the impact of incident delirium on mortality during hospitalization we used a multivariable analysis using a time-dependent Cox regression model to identify the independent factors associated with in-hospital mortality.

The continuous variable distributions were assessed for normality using the Shapiro–Wilk test and for equality of variances using Levene’s test. The data were analyzed using the SPSS v.17 software suite (SPSS Inc., Chicago, IL, USA) and statistical significance level was established at *p* < 0.05.

## 4. Results

### 4.1. Characteristics of Patients

**Figure 1:**
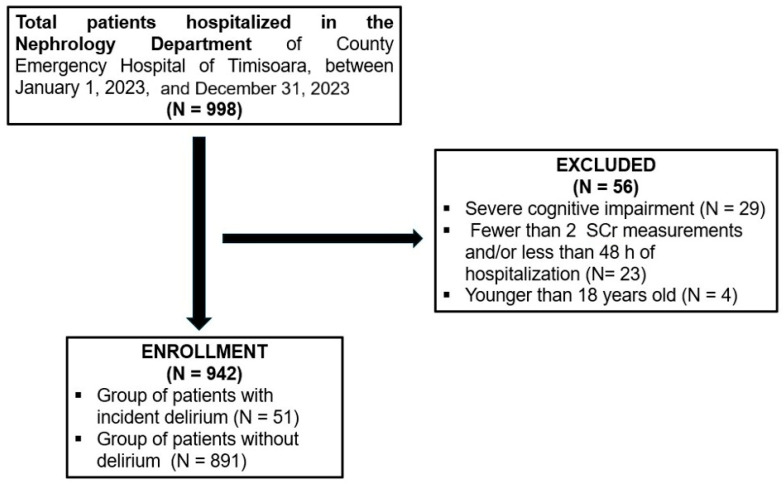
Flowchart of the study.

Between January 2023 and December 2023, a total of 998 patients were admitted to the Nephrology Department of County Emergency Hospital of Timisoara; 29 of them displayed severe cognitive impairment and were not included. Of the 969 remaining patients, 23 individuals had fewer than two SCr measurements during hospitalization and/or were hospitalized for less than 48 h, while 4 subjects were younger than 18 years old and were excluded from the study. Therefore, in this analysis, 942 patients were included (Figure 1).

The baseline socio-demographics and clinical characteristics are presented in Table 1.

The median age of the studied patients was 65 years and 55.1% of subjects were males. During hospitalization, fifty-one patients (5.41%) developed delirium. The median age of patients with incident delirium was significantly higher compared to the non-delirium patients (73 years vs. 65 years, *p* = 0.0003). The patients with delirium exhibited a higher propensity for alcohol consumption (*p* = 0.0004). The patients who developed delirium had more pre-existing neurological and psychiatric diseases, such as a previous stroke (*p* < 0.001) and vascular dementia (*p* = 0.0014). An acute kidney injury was significantly more common in the patients with delirium than in the patients without delirium (62.74% vs. 31.54%, *p* < 0.001). When we analyzed the association between the AKI stage and delirium, we found that moderate-to-severe AKI (AKI stages 2 and 3) was significantly more prevalent among patients with delirium vs. those with no delirium (60.78% vs. 29.18%, *p* < 0.001), whereas AKI stage 1 had a similar prevalence in the two groups (1.96% in delirium group vs. 2.35% in non-delirium group, *p* = 0.855).

Concerning the drug regimens, no patient received medication associated with delirium, such as anticholinergics, opioids, and antihistamines, apart from three patients (0.32%) with severe alcohol withdrawal who required benzodiazepines. The patients with delirium were treated with antipsychotic drugs (haloperidol or quetiapine) in 82.35% of cases, and in 15.7% of cases physical restraints were used.

### 4.2. Predictors of Delirium

Univariable and multivariable logistic regression analyses were conducted to identify independent predictors of delirium among the studied patients. The independent predictors were age (OR = 1.029; 95%CI: 1.002–1.056; *p* = 0.034), history of stroke (OR = 3.493; 95%CI: 1.849–6.598; *p* < 0.001), alcohol abuse (OR = 4.728; 95%CI: 1.968–11.359; *p* = 0.001), and moderate-to-severe AKI (AKI stages 2 and 3) (OR = 2.175; 95%CI: 1.152–4.105; *p* = 0.017) (Table 2).

### 4.3. Outcomes of Patients with Delirium

**Figure 2:**
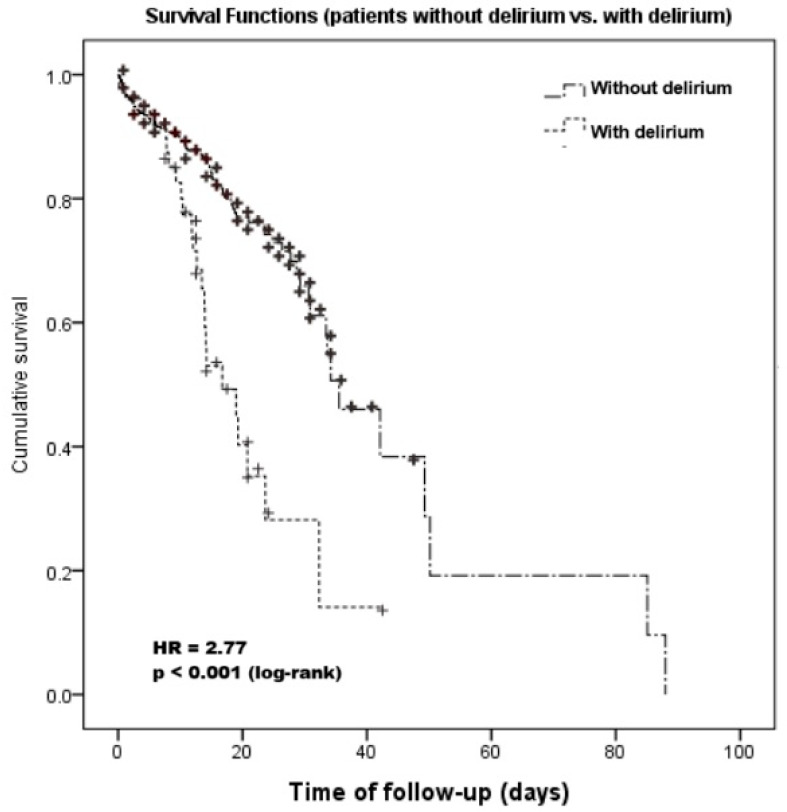
Survival analysis: Patients with vs. without delirium. Kaplan–Meier survival analysis of patients with delirium vs. patients without delirium.

The patients who developed delirium had a significantly longer LOS compared with the patients without delirium (11.96 days vs. 8.86 days, *p* = 0.007). In our cohort, the in-hospital mortality rate was 16.13%. The occurrence of delirium was associated with a significantly higher rate of in-hospital mortality when compared with the non-delirium group (47.05% versus 14.36%, *p* < 0.001). Also, from the time-to-event analysis, delirium was associated with raised mortality (HR = 2.77; 95%CI [1.79 to 4.29]; *p* < 0.001) (Figure 2).

In order to assess the involvement of multiple factors in relation to the death risk during hospitalization, a multiple, backward conditional (stepwise; acceptance threshold, *p* < 0.1; exclusion threshold, *p* > 0.2) Cox proportional hazards model was built, having the following co-factors: age, baseline eGFR, delirium, sepsis, dehydration, previous stroke, heart failure, coronary artery disease, vascular dementia, and AKI. The stepwise algorithm accepted in the Cox model included the following predictors—age, delirium (dichotomous), sepsis (dichotomous), and AKI (dichotomous)—and the resulting model revealed the significant influence of delirium (HR = 1.66; *p* = 0.024), age (HR = 1.032; *p* < 0.001), sepsis (HR = 3.81; *p* < 0.001), and AKI (HR = 1.45; *p* = 0.036) on the risk of in-hospital mortality. The results of the accepted model are presented in Table 3.

## 5. Discussion

In this prospective observational study of hospitalized patients at one academic health center of nephrology in Romania, we found that moderate-to-severe AKI (AKI stages 2 and 3), age, history of stroke, and alcohol consumption were associated with incident delirium in hospitalized patients. Out of the risk factors for delirium, AKI stages 2 and 3 elevated the odds by more than twofold, a history of stroke increased the risk by over threefold, and alcohol misuse raised the risk almost fivefold. The length of hospitalization was about 3 days longer in the delirious compared to the non-delirious patients (11.9 versus 8.8 days, *p* = 0.007). Moreover, delirium was independently correlated with increased in-hospital mortality. These findings indicate that moderate-to-severe AKI is a stronger predictor for incident delirium and suggest that delirium is a significant risk factor for poor short-term outcomes of nephrological patients.

This is the first study to analyze the short-term health outcomes associated with delirium in patients admitted to a nephrology ward.

### 5.1. Prevalence of Delirium

In our study, the rate of delirium occurrence was 5.41%, similar to that reported by another recent study of patients from internal medicine wards in Israel [5]. However, in our cohort, the incidence of delirium was on the lower end of the range that has been observed in most studies [10,11,12,17]. Nonetheless, the significant disparities across the studies are likely related to the type of medical center, the demographic composition of hospitalized patients, and the criteria for selecting the research group. It is important to underscore that our study excluded individuals with significant health issues, such as severe dementia and terminal illnesses. Furthermore, it is possible that some patients with hypoactive delirium were not recognized because this condition can readily be misinterpreted as dementia, depression, or weariness. It is plausible to assume that the patients in these cohorts would exhibit an increased prevalence of delirium.

### 5.2. Risk Factors Associated with Delirium

In this large cohort of nephrological patients, we showed a significant association between moderate-to-severe AKI and delirium, with an odds ratio of 2.175 (95%CI: 1.152 to 4.105). Several prior studies have identified AKI as a key risk factor for delirium. One large observational prospective study involving 1487 neurological patients revealed a significant correlation between AKI and delirium, demonstrated by a markedly increased odds ratio for delirium in individuals with AKI of 10.01 (95%CI: 1.13 to 88.73) [11]. Also, in subjects with gastrointestinal and hepato-pancreato-biliary diseases, acute renal failure was an independent predictor for delirium, increasing the risk of delirium more than fourfold [12].

Though most of the data about the association between AKI and delirium has focused on comparisons of patients with AKI to those without, several studies have highlighted that the association is stronger with higher severity of AKI [7,22,24,32]. A recent study conducted on a critically ill population revealed that among patients with AKI, only higher stages of AKI (AKI stages 2/3) were independently associated with delirium, with an odds ratio of 1.69 (95%CI: 1.04 to 2.73) [32]. Also, another prospective cohort study including ICU patients demonstrated that AKI stages 2 and 3 were correlated with an increased risk for delirium [24]. In addition, in a case–control study conducted on ICU patients, Wan RYY et al. showed that individuals with AKI stage 3 exhibited a fivefold increased likelihood of developing delirium, whereas less severe AKI stages were not significantly correlated with delirium [7]. Another survey of one large cohort of patients undergoing a coronary artery bypass grafting procedure showed that the risk of postoperative delirium increased gradually with the AKI severity [22].

The results of our study are in line with these prior studies. Our findings indicate that the correlation between AKI severity and the occurrence of delirium can be expanded beyond ICUs and surgical patients to include individuals hospitalized in nephrology departments. Also, consistent with the result of previous studies, in this nephrological cohort of patients, we identified a dose-dependent association between AKI severity and delirium. This relationship suggests that delirium might not be totally related to AKI per se, but at the same time, delirium could be due to disturbances of homeostasis induced by AKI, such as volume overload or reduced clearance of some neurotoxins or medications and their metabolism [22,33].

Currently, it is known that AKI induces dysfunction of distant organs, not only impairing the brain, but also the liver, heart, and lungs [34]. The pathophysiology of AKI-associated delirium is multifactorial and complex, including both inflammatory and non-inflammatory mechanisms. AKI generates systemic inflammation and impaired cytokine clearance, which is associated with structural brain lesions [33]. In patients with delirium, it was demonstrated that pro-inflammatory cytokines, such as IL-6, IL-1α, IL-1β, and TNF-α, are associated with delirium-like behavioral disturbances, such as diminished concentration, altered motivation, and decreased psychomotor activities [35]. Moreover, the accumulation of uremic toxins and drugs increases the permeability of the blood–brain barrier. Also, fluid overload related to AKI can induce blood–brain barrier leakage, resulting in brain vasogenic edema with a raised risk of delirium [33]. In addition, AKI-associated disequilibrium of hormonal balance and of neurotransmitter turnover may be related to delirium development [33].

Our study found that age was significantly associated with risk of delirium among nephrological patients. This finding is consistent with the data from the literature demonstrating that older age is an independent risk factor for delirium among elderly, medically ill, and surgical patients [11,13,22,32,36,37]. Advanced age is likely a risk factor for the onset of delirium due to a heightened prevalence of medical comorbidities; increased overall frailty; diminished number of acetylcholine-producing neurons; exacerbated cognitive impairments; age-related alterations in stress-regulating neurotransmitters, and chronic neurodegeneration accompanied by augmented production of inflammatory mediators [36].

Alcohol misuse was an additional risk factor for delirium in our investigation, in line with the results reported by two previous studies involving critically ill patients [7,32]. In our study we found that a history of stroke was a significant risk factor for delirium. This association could be due to vascular cognitive impairment after a stroke that leads to an increased susceptibility to delirium [36]. Similar findings were reported by two other recent studies [13,37].

### 5.3. Outcomes Associated with Delirium

In our study we showed that nephrological patients with delirium had substantially longer LOSs. This data is consistent with the results reported by other studies conducted on specific patient cohorts, such as elderly, ICU, and surgical patients [7,20,23,32,37,38].

A large-scale survey analyzed data on older adults across 45 UK acute care hospitals and found that delirium increased the LOS by an average of 3.45 days [20]. Two recent studies performed on patients with AKI treated in ICUs demonstrated that delirium was independently associated with longer ICU stays [7,32]. Moreover, among neurosurgery patients, those with delirium had a 1-week-longer hospitalization compared to non-delirium individuals [37]. In one systematic review and meta-analysis, Salluh et al. showed that critically ill patients with delirium had a significantly longer LOS compared to non-delirium subjects [6]. Another recently published meta-analysis of 41 unique studies that included 117,255 patients found that delirium increased the LOS by nearly one week and generated significantly higher costs [38]. Individuals with delirium frequently require complex care interventions and need more time to achieve a mental and physical condition that allows for discharge from the hospital, thus leading to significantly longer LOSs [38].

To date, delirium is widely recognized to be associated with increased in-hospital mortality [6,9,10,11,12,20,21,22,23]. Moreover, the in-hospital risk of mortality associated with delirium has not improved over the past thirty years, despite the progress in delirium research [9].

Concerning the relationship between delirium and mortality during hospitalization, previous studies have mainly focused on older, surgical and ICU-admitted patients [6,9,20,21,22,23], whereas a very few studies have analyzed the short-term outcomes of delirium in individuals admitted to internal medicine settings [10,11,12,13]. Furthermore, the reports about delirium and its associated risk of mortality among patients with kidney disease are limited to only two studies that included patients with end-stage kidney disease undergoing hemodialysis [25,26].

For the first time, our study found a significantly higher in-hospital mortality rate among nephrological patients with delirium compared to non-delirium subjects, with almost half of patients with delirium dying in the hospital. These findings are consistent with the data from the literature that have revealed a wide range of rates of in-hospital delirium-related mortality that vary from 6.7% up to 63.6% [21]. However, compared to other research that has evaluated the mortality associated with delirium among patients hospitalized in general medical wards, such as gastroenterology [12] or neurology units [11], our study revealed a higher rate of in-hospital mortality. Thus, in our nephrological patients with delirium, the in-hospital mortality rate was 47.05%, whereas in patients with gastrointestinal and hepato-pancreato-biliary diseases with delirium the rate was 18% [12], and in neurological patients with delirium the rate was 5.9% [11]. We may assume that in patients with kidney diseases, the delirium-associated risk of death could be linked to the complexity of underlying medical conditions or to the disease’s severity, with delirium being only a marker of illness severity.

Furthermore, in our research, the time-to-event analysis regarding the in-hospital mortality highlighted the negative impact of delirium upon the in-hospital survival for nephrological patients, with a hazard ratio of 2.77. In addition, the multivariable regression analysis confirmed that delirium was independently associated with increased in-hospital death. Recently, one of the largest published meta-analyses demonstrated a significant correlation between delirium and mortality [9]. This report, with over 49,566 participants, showed that elderly subjects with delirium had a more than three times greater rate of mortality compared to non-delirious subjects. Moreover, critically ill patients experiencing delirium in the ICU had the highest odds for in-hospital mortality (OR: 7.09; [95%CI: 3.60, 14.0]), representing a twofold increase in risk relative to the average [9]. In one longitudinal observational study including an acute care patient cohort, delirium was an independent predictor of mortality, with a crude OR of 5.46 for the risk of death [8]. Moreover, delirium was an independent predictor of mortality in surgical wards [22,23] and in internal medicine settings, such as gastroenterology [12], neurology [11], and medical oncology and hematology departments [39].

The pathophysiology of the relationship between delirium and mortality are partially understood. Delirium per se is associated with overactivation of microglia and an aberrant stress-induced response with consequent neurotransmitter disturbances, uncontrolled neuroinflammation, and cerebral metabolism alterations [36,40]. These pathophysiological processes accentuate acute brain failure, which may further lead to distant organ dysfunctions [36,40,41]. Delirium may also increase mortality indirectly due to its negative consequences, such as prolonged hospitalization with its higher risk of hospital-acquired infections, dehydration, sleep deprivation, aspiration pneumonia, or the use of physical restraints methods [42].

Given the prevalence and adverse consequences of delirium, our results underscore the need for prospective cohort studies with standardized methods to accurately and reliably detect and rate delirium and to characterize its short- and long-term outcomes. Such studies need to be stringent in identifying all factors that could contribute to the onset, pathogenesis, and resolution of delirium associated with critical illness.

The findings of our study have practical implications and emphasize the necessity of enhanced efforts for identifying delirium among patients admitted to a nephrology unit. This research suggests that subjects with AKI stages 2 and 3, alcohol abuse, a history of stroke, or older patients are the most vulnerable to developing delirium. This study demonstrates that nephrological patients with delirium represent a subgroup with prolonged hospitalization and with a higher risk for death during admission. Identifying these patients in the hospital may create an opportunity to increase awareness of delirium and its possible implications among patients and nephrologists. Our findings underline the need for prospective cohort studies utilizing standardized instruments for accurately diagnosing delirium and to delineate the risk factors for delirium, as well as the short- and long-term outcomes among nephrological patients.

### 5.4. Strengths and Limitations

For the first time, this study assessed the risk factors for delirium in a large cohort sample of patients admitted to a nephrology ward at a tertiary care center. Moreover, to the best of our knowledge, this is the first report about the short-term health outcomes associated with delirium in nephrological patients. The relevant medical and demographic variables, together with comorbidities, were recorded. Furthermore, we used the definition of AKI according to the KDIGO criteria, which is highly sensitive and classifies minor and transient increases in creatinine as AKI. The limitations comprise its observational design and restriction to a single center, thus limiting the generalizability of the findings and excluding any assumption about a causal relationship. In addition, we identified the presumed delirious patients according to their clinical signs and delirium was confirmed by a psychiatric evaluation. Our strategy for identifying delirious patients might have been less sensitive and underestimated the delirium rate, given that patients with hypoactive delirium often receive diminished clinical attention and their delirious characteristics are more challenging to recognize. Prior advanced dementia may co-exist with delirium. Nevertheless, we excluded individuals with a prior severe cognitive impairment that made verbal communication impossible, resulting in an underestimated prevalence of delirium. Further, not all the risk factors reported in the literature [36] were measured (e.g., environmental factors, such as sensory deprivation or isolation; use of physical restraints or immobility; etc.).

## 6. Conclusions

To conclude, though the fact that the prevalence of delirium in our research was on the lower range of that reported in the literature for internal medicine hospitalized populations, our results remain alarming, mainly concerning the negative outcomes associated with this neuropsychiatric syndrome among nephrological patients. In our study, delirium was a severe condition correlated with prolonged hospitalization and with an increased mortality rate. The present study highlights that AKI stages 2 and 3, older age, alcohol misuse, and previous stroke were significant risk factors for delirium in nephrological patients, suggesting that prevention strategies must be focused mainly on these categories of patients. Nephrology departments must promote early screening for delirium and the best preventive strategies to improve patients’ outcomes and to reduce costs.

## Figures and Tables

**Table 1:** Demographic, clinical, and biological data of the patients and a comparison between the patients with delirium vs. patients without delirium.

Parameters			Total Group (n = 942)	Delirium (n = 51)	No Delirium (n = 891)	*p*
Age [IQR], years			65 [20]	73 [14]	65 [20]	0.0003 *
Men, n (%)			519 (55.1)	32 (62.74)	487 (54.65)	0.311
Smoking, n (%)			38 (4.03)	4 (7.84)	34 (3.81)	0.144
Alcohol misuse, n (%)			45 (4.77)	9 (17.64)	36 (4.04)	0.0004*
Serum creatinine [IQR], (mg/dL)			4.34 [4.99]	5 [4.6]	4.33 [4.99]	0.275
eGFR [IQR], (ml/min/1.73m^2^)			11.88 [24.51]	10.49 [19.58]	11.98 [24.93]	0.179
Comorbidities, n (%)	Arterial hypertension, n (%)		833 (88.43)	47 (92.15)	786 (88.21)	0.528
	Diabetes mellitus, n (%)		373 (39.60)	20 (39.21)	353 (39.62)	0.954
	Coronary artery disease, n (%)		361 (38.32)	24 (47.06)	337 (37.82)	0.235
	Heart failure, n (%)		352 (37.37)	24 (47.06)	328 (36.81)	0.179
	History of stroke, n (%)		124 (13.16)	20 (39.21)	104 (11.67)	<0.001 *
	Vascular dementia, n (%)		53 (5.62)	9 (17.64)	44 (4.94)	0.0014 *
	Chronic kidney disease, n (%)		767 (81.42)	37 (72.55)	730 (81.93)	0.097
	Peripheral vascular disease, n (%)		46 (4.88)	4 (7.84)	42 (4.71)	0.306
	Dyslipidemia, n (%)		315 (33.44)	10 (19.60)	305 (34.23)	0.032
	Cirrhosis, n (%)		39 (4.14)	1 (1.96)	38 (4.26)	0.717
	Chronic obstructive pulmonary disease, n (%)		72 (7.64)	5 (9.80)	67 (7.52)	0.583
	Depression, n (%)		83 (8.81)	8 (15.68)	75 (8.42)	0.120
Acute kidney injury, n (%)			313 (33.22)	32 (62.74)	281 (31.54)	<0.001 *
AKI stage		AKI stage 1, n (%)	22 (2.44)	1 (1.96)	21 (2.35)	0.855
		AKI stages 2 and 3, n (%)	291 (30.90)	31 (60.78)	260 (29.18)	<0.001 *
Length of hospital stay [IQR], days			9 [10.59]	11.96 [9.62]	8.88 [10.57]	0.007 *
In-hospital mortality rate, n (%)			152 (16.13)	24 (47.05)	128 (14.36)	<0.001 *

* Predictor is significant.

**Table 2:** Results of univariable and multivariable analyses of risk factors for delirium.

Parameter	Univariate Analysis			Multivariate Analysis		
	Beta	OR (95%CI)	*p*	Beta	OR (95%CI)	*p*
Age (years)	0.042	1.043 (1.019 to 1.068)	<0.001	0.028	1.029 (1.002 to 1.05)	0.034
AKI stages 2 and 3 KDIGO	1.325	3.762 (2.105 to 6.721)	<0.001	0.777	2.175 (1.152 to 4.105)	0.017
Alcohol misuse	1.627	5.089 (2.302 to 11.252)	<0.001	1.553	4.728 (1.968 to 1.359)	0.001
Dyslipidemia	0.758	0.469 (0.232 to 0.948)	0.035	-	-	-
Vascular dementia	1.417	4.125 (1.889 to 9.01)	<0.001	-	-	-
History of stroke	1.586	4.882 (2.684 to 8.89)	<0.001	1.251	3.493 (1.849 to 6.598)	<0.001

**Table 3:** Predictors of in-hospital mortality accepted in the Cox proportional hazards model.

Predictor	B	Wald	Hazard Ratio [95%CI]	*p*
Age	0.032	18.322	1.032 [1.017–1.047]	<0.001 *
Baseline eGFR	−0.008	2.605	0.992 [0.982–1.002]	0.107
Delirium	0.511	5.080	1.666 [1.069–2.597]	0.024 *
Sepsis	1.338	43.449	3.811 [2.560–5.673]	<0.001 *
AKI	0.374	4.375	1.453 [1.024–2.062]	0.036 *

* Predictor is significant.

## Data Availability

The data that support the findings of this study are available from the corresponding author upon reasonable request.
